# Increased MACC1 levels in tissues and blood identify colon adenoma patients at high risk

**DOI:** 10.1186/s12967-016-0971-0

**Published:** 2016-07-20

**Authors:** Hassan Ashktorab, Pia Hermann, Mehdi Nouraie, Babak Shokrani, Edward Lee, Tahmineh Haidary, Hassan Brim, Ulrike Stein

**Affiliations:** Department of Medicine and Cancer Center, Howard University, 2041 Georgia Avenue NW, Washington, DC 20059 USA; Experimental and Clinical Research Center, Charité University Medicine Berlin and Max-Delbrück-Center for Molecular Medicine, Robert-Rössle-Straße 10, 13125 Berlin, Germany; Department of Pathology, Howard University, 2041 Georgia Avenue NW, Washington, DC 20059 USA; German Cancer Consortium, Im Neuenheimer Feld 280, 69121 Heidelberg, Germany

**Keywords:** Colorectal cancer, Adenoma, Prognosis, MACC1

## Abstract

**Background:**

Colorectal cancer is a preventable disease if caught at early stages. This disease is highly aggressive and has a higher incidence in African Americans. Several biomarkers and mutations of aggressive tumor behavior have been defined such as metastasis-associated in colon cancer 1 (MACC1) that was associated with metastasis in colorectal cancer patients. Here, we aim to assess colon tissue MACC1 protein and circulating MACC1 transcripts in colon preneoplastic and neoplastic African American patients.

**Methods:**

Patients’ tissue samples (n = 143) have been arranged on three tissue microarrays for normal (n = 26), adenoma (n = 68) and cancer (n = 49) samples. Immunohistochemistry was used to detect MACC1 expression. Blood samples (n = 93) from normal (n = 45), hyperplastic (n = 15) and tubular adenoma (n = 33) patients were used to assess MACC1 transcripts using qRT-PCR. Distribution of continuous variables was tested between different diagnoses with Kruskal–Wallis test. Categorical variables were tested by Chi square test. We assessed the prognostic ability of IHC staining by calculating area under receiver operating characteristics curve (ROC) for adenoma and cancer separately. Differences between groups in terms of MACC1 transcript levels in plasma were calculated by using non-parametric (exact) Wilcoxon-Mann–Whitney tests. We performed all calculations with SPSS, version 21.

**Results:**

In patient tissues, there was a statistically significant difference in MACC1 expression in normal vs. adenoma samples (p = 0.004) and normal vs. cancer samples (p < 0.001). There was however no major difference in MACC1 expression between adenoma vs. cancer cases or tubular adenomas vs tubulovillous adenomas. The area under the curve for both normal vs. adenoma and normal vs. cancer cases were 70 and 67 %, respectively. MACC1 expression was not correlated to age, gender or anatomical sample location. In patient plasma, MACC1 transcripts in adenoma patients were significantly higher than in plasma from normal patients (p = 0.014). However, the difference between normal and hyperplastic plasma MACC1 transcripts was not statistically significant.

**Conclusion:**

Metastasis-associated in colon cancer 1 is expressed at early stages of colorectal oncogenesis within the affected colonic tissue in this patient cohort. The plasma transcripts can be used to stratify African American patients at risk for potential malignant colonic lesions.

## Background

Colorectal cancer (CRC) is the second most common cancer causing mortality in Western world [1]. Its incidence is high in African Americans when compared to the general population [2–9]. African Americans also display very advanced and aggressive forms of CRC. There is a need to analyze these patients’ tumors for markers of such aggressive/advanced pathology [4–6, 8, 10, 11].

The novel gene MACC1 (metastasis associated in colon cancer 1) was identified by our group in human CRC [12]. Following our initial publication, evidence was provided that MACC1 regulates fundamental processes like proliferation, migration, invasion, and dissemination in cell culture by regulating genes important e.g. for metastasis [12–16]. Subcutaneous, orthotopic, and intrasplenic transplantation of MACC1-expressing tumor cells induced tumor growth and metastasis in mice, shRNA acting on MACC1 or MACC1 targets decreased metastases [12, 17, 18]. First transgenic MACC1 mice crossed with Apc^Min^ mice demonstrated an accelerated adenoma-carcinoma sequence [19]. In CRC patients, MACC1 is a tumor stage-independent predictor for metastasis and survival [20–23].

Metastasis associated in colon cancer 1 has been established as a prognostic biomarker for a further variety of solid cancers such as the gastrointestinal tract (CRC, gastric, pancreatic), hepatobiliary, lung, ovarian, breast, renal, nasopharyngeal, esophageal, kidney, bladder, gallbladder cancers, to glioblastomas and osteosarcomas [24–27].

Expression of MACC1 correlates to tumor formation, metastases and patient survival, determined in cryo and formalin fixed paraffin embedded normal, tumor and metastatic tissues from retrospective and prospective studies. We also established the detection of circulating MACC1 transcripts in cancer patient blood [28, 29]. In prospective studies we demonstrated significant correlations of circulating MACC1 transcripts in colon, rectal and gastric cancer patients’ plasma with patients’ survival. Correlations of circulating MACC1 transcripts and MACC1 protein expression with survival for lung and pancreatic cancer patients were also reported [30, 31].

Since clinical and histopathological classifications as well as current tissue-based molecular markers do not provide enough information for an early and precise identification of patients at high-risk for aggressive tumor progression and metastasis formation, it is of upmost importance to define molecular biomarkers, which will allow the identification of high-risk patients at early stages of the disease. MACC1 was found elevated at the crucial transition step from adenoma to carcinoma as well as in early stages of the disease allowing the early identification of high-risk patients [12, 32, 33].

Here we aimed to identify the diagnostic and prognostic potential of MACC1 levels for African American patients suffering from colon lesions in different stages using immunohistochemistry (IHC) on tissues. We also aimed at a non-invasive determination of circulating MACC1 transcripts in the plasma of these patients by quantitative real time reverse transcriptase-polymerase chain reaction (qRT-PCR). For adenoma patients, such a MACC1-based blood test was never employed before with respect to diagnose adenoma cancer patients vs. lesion-free volunteers. We hypothesize that analyzing the MACC1 levels in liquid biopsies of African American adenoma patients might be beneficial for prognosis of the disease. We demonstrate significant correlations of MACC1 levels to early and late stage adenomas reflecting the potential use of this marker as a surrogate for tumor progression and metastasis risk assessment.

## Methods

### Aim, design and setting of the study

We aimed to identify the diagnostic and prognostic potential of MACC1 levels for African American patients suffering from colon lesions in different stages. We assessed MACC1 protein using IHC on patient tissues samples arranged on microarrays as well as qRT-PCR for circulating transcripts in the plasma of these patients.

### Tissue specimens, tissue microarrays (TMAs), and IHC

Samples were recruited from pathology department at Howard University Hospital. All specimens were collected after written informed consent in accordance with the International Conference on Harmonization. The study was approved by The Howard University Institutional Review Board (IRB-06-MRF-39). All patients signed a consent form to participate in the study and to have the data published without identifiers.

The TMA sections were made from all duplicated samples and put in three different TMA slides as normal matched (n = 26), adenomatous (n = 68) and adenocarcinoma (n = 49). A total of 143 samples were stained for MACC1 by IHC using an anti-MACC1 antibody. Briefly, the TMA sections were deparaffinized by successive immersions in xylene (20 min), acetone/Tris 2:1, acetone/Tris 1:2, Tris/NaCl, aqua dest (5 min each). Epitopes were demasked with 10 mM citrate buffer (pH 6, microwave). After blocking (5 % goat serum, 30 min), sections were incubated with the rabbit polyclonal anti-MACC1 antibody (1:50, Sigma HPA020103) for 3 h at room temperature. Detection was performed using the biotin-based ABC kit (Dako; anti-rabbit biotin antibody and anti-biotin-streptavidin-HRP) and diaminobenzidine (1 min) as substrate. Counter staining with Mayers haematoxylin was done for 1 min. Negative biological controls were performed using non colon tissues as well as duplicates of the TMAs’ colon samples to assess reproducibility of the results with each given sample. Negative technical controls were carried out by omitting the primary MACC1 antibody. The stained TMAs were read by two pathologists including one gastrointestinal pathologist (E.L). The protein expression was reported as staining 0, 1, 2, 3, 4 which refer to none, less than 10, 10–25, 25–50, 50–75, >75 %, respectively. Sub-cellular staining was reported as nuclear, cytoplasmic, or both.

### Plasma specimens

Blood specimens from patients and lesion-free volunteers were collected after written informed consent in accordance with the International Conference on Harmonization. The study was approved by The Howard University Institutional Review Board (IRB-06-MRF-39). All patients signed a consent form to participate in the study and to have the data published without identifiers.

We collected 45 blood samples of healthy colon lesion-free patients and 48 blood samples of patients with colonic lesions consisting of 33 tubular adenomas (TA) and 15 hyperplastic polyps (HPP). All these patients were diagnosed at Howard University Hospital, Washington DC, from 2011 until 2013. Patients’ data was collected from medical records and pathology reports. Exclusion criteria were history of cancer of any type. Blood samples of the included patients were taken at the day of diagnosis.

### Plasma preparation

Plasma separation was performed from cooled EDTA-blood at the same day within 7 h post blood taking. This was performed on normal (n = 45), TA (n = 33) and HPP patients’ blood (n = 15). The freshly collected blood samples were centrifuged at 1300 rpm for 10 min at 10 °C. The supernatant was again centrifuged at 2500 rpm for 15 min and 4 °C to remove all cell debris. The supernatant (plasma) was pipetted into new Eppendorf tubes and stored at −80 °C. Samples were blinded so that neither tumor entity nor disease stage was disclosed during analysis.

### qRT-PCR

Isolation of total RNA and qRT-PCR were performed as previously described [23]. Briefly, after 30 s at 95 °C, we run 45 cycles of 10 s 95 °C, 10 s 62 °C, 10 s 72 °C, and a melting curve from 40 °C to 95 °C, using the LightCycler (DNA Master HybProbe kit, Roche Diagnostics). Thereby we amplified a 136 bp MACC1-specific PCR product with the following primers and probes: forward primer 5′-TTCTTTTGATTCCTCCGGTGA-3′, reverse primer 5′-ACTCTGATGGGCATGTGCTG-3′, FITC-probe 5′-GCAGACTTCCTCAAGAAATTCTGGAAGATCTA-3′, LCRed640-probe 5′-AGTGTTTCAGAACTTCTGGACATTTTAGACGA-3′ (syntheses of primers and probes: BioTeZ and TIB MolBiol, Berlin, Germany). The calibrator cDNA derived from the cell lines SW620 (authentication of the cell line by short tandem repeat (STR) genotyping, German Collection of Microorganisms and Cell Cultures, Braunschweig, Germany).

Metastasis-associated in colon cancer 1 mRNA expressions are given as percentage of the mRNA expression of a calibrator sample, which was set 100 %. Each sample was run and calculated in duplicate, the means are depicted.

### Statistical analyses

Distribution of continuous variables was tested between different diagnoses with Kruskal–Wallis test and categorical variables were tested by Chi square test. The agreement between two raters for cytoplasmic percentage (C %) staining was measured by calculating the Intraclass Correlation Coefficient (ICC). We assessed the prognostic ability of IHC staining by calculating area under receiver operating characteristics curve (ROC) for adenoma and cancer separately.

Differences between groups in terms of MACC1 transcript levels in plasma were calculated by using non-parametric (exact) Wilcoxon-Mann–Whitney tests (because of deviations of the distributions from normality and small samples). Samples obtained from lesion-free volunteers were compared with those from patients with adenomas. P values <0.05 were considered to be significant. We performed all calculations with SPSS, version 21.

## Results

### MACC1 immunohistochemistry discriminates between colorectal lesions and normal tissue

One hundred forty-three samples were assessed. Among them 26 (18 %) were normal, 68 (48 %) adenomas (30 tubulovillous (TV) and 38 TA) and 49 (34 %) were CRC. Table 1 indicates the demographic and clinical characteristics of these samples.

**Table 1:** Distribution of demographic and clinical characteristics by diagnosis

	Normal	Adenoma	CRC	P value
Male gender, no (%)	12 (46 %)	38 (56 %)	23 (48 %)	0.6
Age, median (IQR)	62 (52–74)	59 (52–64)	67 (54–76)	0.004
Age ≥60 years, no (%)	16 (62 %)	27 (43 %)	30 (64 %)	0.06
Location	NA			0.07
Right colon		37 (54 %)	18 (38 %)	
Left colon		31 (46 %)	30 (63 %)	
Mass size, median (IQR)	NA	1.5 (1.3–2.0)	4.0 (2.5–5.5)	<0.001

**Fig. 1:**
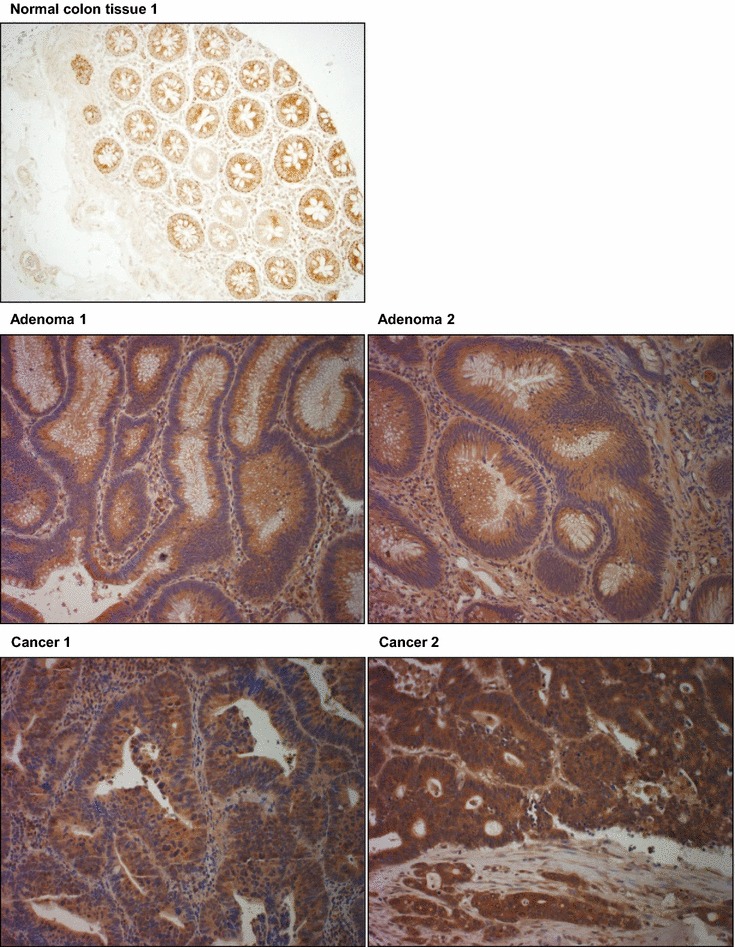
MACC1 TMA IHC of patients’ tissue samples. Representative MACC1 IHC show cytoplasmic staining for normal colon tissue (intensity = 1), for two adenomas (intensity = 2), and for two colorectal cancer samples (Cancer 1: intensity = 3 and Cancer 2, intensity = 4). MACC1 protein expression levels were higher in adenoma samples vs. normal tissues (p = 0.004), and were higher in cancer samples vs. normal tissues (p < 0.001). No major differences in MACC1 expression levels were found between adenoma vs. cancer cases or tubular adenomas vs tubulovillous adenomas. Sections treated without the primary antibody served as controls. Images are presented for 20× magnification

**Fig. 2:**
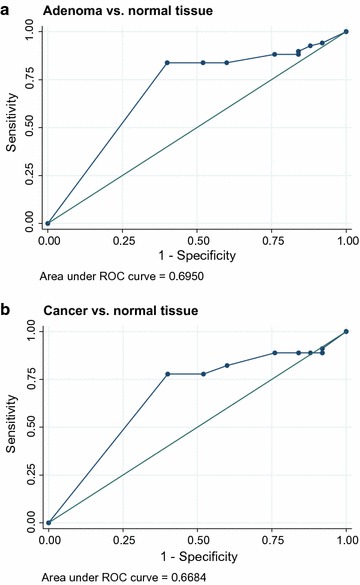
MACC1 cytoplasmic percentage (C %) staining to diagnose between adenoma vs. normal and cancer vs. normal tissues. Both adenoma and CRC samples had significantly higher staining percentages compared with normal tissue. Adenoma vs CRC cases had similar MACC1 staining (ROC (95 % CI) 0.47 (0.40–0.55, not shown). **a** Staining % had a area under ROC curve of 70 % (95 % CI 59–80 %) to diagnose between adenoma and normal samples with highest diagnostic ability in 100 % staining (with Sensitivity = 0.84 and Specificity = 0.60). **b** Staining % had a area under ROC curve of 67 % (95 % CI 59–80 %) to diagnose between CRC and normal samples with highest diagnostic ability in 100 % staining (with Sensitivity = 0.78 and Specificity = 0.60)

Adenoma cases were younger (p = 0.004) and tumor size was larger in CRC (<0.001). Two independent pathologists (E.L, B.S) reviewed the IHC. Representative MACC1 stainings are shown for adenomas, non-metastasized and metastasized CRC (Fig. 1). The ICC for % staining between two reviewers was 0.88 (95 % CI 0.83–0.92) indicating very strong agreement. So we used the reading from first pathologist for further analysis. The distribution of staining in three groups of samples were determined (Table 2). Both adenoma and CRC samples had significantly higher staining percentages compared with normal tissue. Further analysis showed that staining percentage had a area under ROC curve of 70 % (95 % CI 59–80 %) to diagnose between adenoma and normal samples with highest diagnostic ability in 100 % staining (with Sensitivity = 0.84 and Specificity = 0.60) (Fig. 2a). Staining % had a area under ROC curve of 67 % (95 % CI 59–80 %) to diagnose between CRC and normal samples with highest diagnostic ability in 100 % (with Sensitivity = 0.78 and Specificity = 0.60) (Fig. 2b). In all three different groups, staining % was not related to gender or age. In adenoma and CRC, staining % was not related to anatomic location or mass size. A subgroup analysis in adenoma samples, indicated that there was no significant difference between TV and TA with regard to age, gender, tumor location and size as well as IHC measures.

**Table 2:** Distribution of cytoplasmic percentage (C %) staining by diagnosis

	Normal	Adenoma	CRC	P value
C % staining, median (IQR)	95 (80–100)	100 (100–100)	100 (100–100)	<0.001
Positive intensity, no (%)	23 (92 %)	64 (94 %)	41 (89 %)	0.6

### Elevated circulating MACC1 transcript levels in precancerous stages

**Fig. 3:**
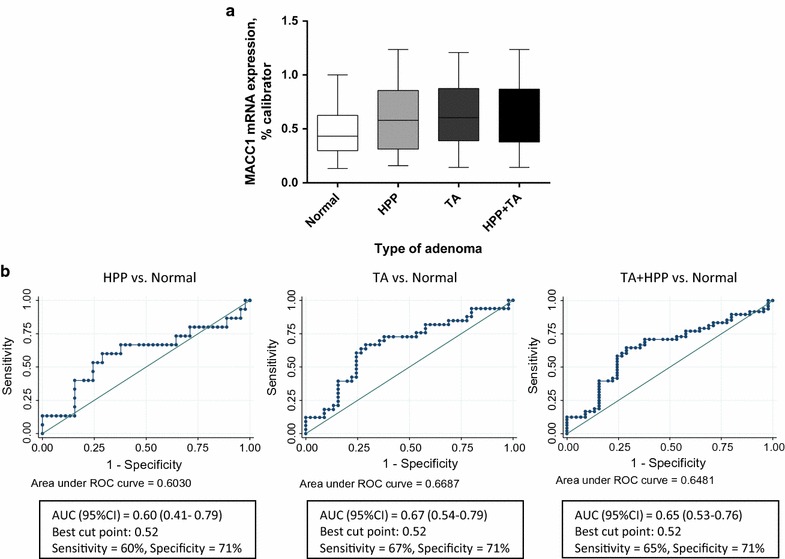
MACC1 transcripts in normal, hyperplastic (HPP) and tubular adenoma (TA) patients’ plasma. **a** MACC1 transcripts were detected in all measured normal (n = 45), HPP (n = 15) and TA (n = 33) patients’ plasma. MACC1 transcript levels were higher in all adenoma patients’ plasma (median 0.6036 MACC1 mRNA expression/percent calibrator) vs. normal patients’ plasma (median 0.4315 MACC1 mRNA expression/percent calibrator; p = 0.014). MACC1 transcript levels were also higher in TA patients plasma (median 0.6045 MACC1 mRNA expression/percent calibrator) vs. normal patients’ plasma (p = 0.011). However, MACC1 transcript levels were not significantly higher in HPP patients plasma (median 0.5788 MACC1 mRNA expression/percent calibrator) vs. normal patients’ plasma (p = 0.239). **b** Area under the curve was calculated for the diagnostic value of plasma MACC1 transcripts for Normal vs. HPP, Normal vs. Adenoma and for Normal vs. Adenoma/HPP combined

In order to test for potential difference in levels of circulating MACC1 transcripts in lesion-free-volunteers and adenoma patients, we measured MACC1 transcripts in normal (n = 45), hyperplastic (n = 15) and tubular adenoma (n = 33) patients’ blood. MACC1 transcripts were detected in all analyzed samples. MACC1 transcripts were detected at a significantly higher level in all adenoma patients’ plasma (median 0.6036 MACC1 mRNA expression/percent calibrator) when compared to plasma of lesion-free volunteers (median 0.4315 MACC1 mRNA expression/percent calibrator; p = 0.014). We then tested for MACC1 levels in different stages of adenoma development. When comparing the normals’ MACC1 transcripts to the group of tubular adenoma patients (n = 33; median 0.6045 MACC1 mRNA expression/percent calibrator), we also found significantly higher values for the adenoma patient group (p = 0.011). However, the difference between normals (median 0.4315 MACC1 mRNA expression/percent calibrator) and hyperplastic patients’ plasma (n = 15; median 0.5788 MACC1 mRNA expression/percent calibrator), MACC1 was not statistically significant. This might be due to the relatively small patient number in this group (p = 0.239) (Fig. 3a), but might also reflect that levels of circulating MACC1 transcript might increase during the course from early to late (HPP to TA) adenomas. This has to be validated in future analyses employing independent patient cohorts. We observed no significant variations of MACC1 levels due to age or sex. Taken together, the levels of circulating MACC1 transcripts in plasma of at least patients with tubular adenoma are higher than in lesion-free volunteers and might indicate those patients at high risk for tumor progression.

We further established the plasma MACC1 transcripts’ diagnostic value using a ROC based analysis. For all comparisons, the best cut point was 0.52. When comparing normal to HPP/adenoma combined, the AUC (95 % CI) was 0.65 (0.53–0.76) with a sensitivity of 65 % and a specificity of 71 %. For adenoma vs. normal, the AUC (95 % CI) was 0.67 (0.54–0.79 with a sensitivity of 67 % and a specificity of 71 %. These values were AUC (95 % CI): 0.60 (0.41–0.79 with a sensitivity of 60 % and a specificity of 71 % for the HPP vs. normal comparison (Fig. 3b).

## Discussion

Many studies attempted to identify and detect biomarkers of CRC and its precursor lesions in non-invasive manners that would not require the use of colonoscopy or other invasive procedures [34–39]. The present study is an attempt in that direction in a population that is known to suffer a higher burden of aggressive CRC than the general population [4–6, 9, 10, 40–42]. The identification of high-risk patients in the pre-malignant stage of adenoma by non-invasive technology would potentially contribute to early detection and possible intervention to alter the process of tumor progression.

Our tissue microarray analysis of normal, adenoma, and cancer samples revealed that MACC1 staining was not related to gender or age, nor was it related to anatomic location or mass size of adenoma or cancer lesions. MACC1 immunohistochemical staining was significantly different between normal vs. adenoma, and between normal vs. cancer cases, the p values for these differences were 0.004 and <0.001, respectively. These findings reflect a direct relationship of MACC1 expression with the neoplastic transformation at early stages with MACC1 being involved in processes other than metastasis alone. Indeed, MACC1 was shown to be involved in many processes such as proliferation, tumor-formation ability, migration and invasiveness that might begin at pre-cancerous stages. This is underlined by the difference in p values between cancer vs. normal (<0.001) and adenoma vs. normal cases (p = 0.004). MACC1 immunohistochemical staining differences between adenoma and cancer cases were not statistically significant, In line, there was also no significant difference in MACC1 staining between tubular adenoma and tubulovillous adenomas (advanced aggressive histology adenoma). This finding points likely to an early onset of expression induction of MACC1 once the neoplastic process is launched in this cohort of African American patients. It is noteworthy that the adenoma cases were younger (p = 0.004) and their lesions smaller in size when compared to CRC (<0.001), but still MACC1 could be detected in these specimens at statistically significant levels compared to normal tissues. Already at this stage, we notice a potential use of this marker to detect neoplastic lesions with potential metastatic features. Indeed, further analysis of the staining results led to an area under ROC curve of 70 % (95 % CI 59–80 %) to diagnose between adenoma and normal samples with highest diagnostic ability in 100 % staining (with Sensitivity = 0.84 and Specificity = 0.60) (Fig. 2) while the area under ROC curve was 67 % (95 % CI 59–80 %) to diagnose between CRC and normal samples with highest diagnostic ability in 100 % (with sensitivity = 0.78 and specificity = 0.60) (Fig. 2).

While most of the published data on MACC1 expression was done in cancer specimens, our study here in precancerous lesions highlights important roles of MACC1 at earlier stages. We previously reported significantly MACC1 higher levels in tumors and blood of early stages CRC patients compared to normal mucosa or healthy volunteers, respectively [12, 28]. But although we also found MACC1 levels in adenomas there was not a statistically significant difference when compared to normal mucosa in the analyzed Caucasian cohort [12]. Ren and colleagues published a stepwise elevation of MACC1 expression in key points of CRC development (colorectal adenoma, early-stage invasive and advanced adenocarcinoma with liver metastasis) by IHC suggesting that MACC1 may contribute to cancer initiation and early invasive growth [33]. However, no comparison of MACC1 levels to those of healthy volunteers was shown.

Here we addressed the identification of high-risk patients at pre-malignant stages of adenoma focused on the African American population known to suffer from aggressive CRC. Thus, our findings not only point to a role of MACC1 in oncogenesis within the affected colonic tissue, but might also be of benefit for at risk patients’ population.

These findings on patient tissue prompted us to further analyze MACC1 expression at the transcript level in blood samples of precancerous colorectal patients. To further pinpoint specific occurrence of MACC1 in patients’ bloodstream, we included samples from normal, hyperplastic and tubular adenoma patients. MACC1 transcripts were detected at a significant higher level in the combined group of hyperplastic and tubular adenoma patients’ plasma when compared to lesions-free patients’ plasma (p = 0.014). Significance was also reached when comparing lesion-free patients’ MACC1 transcripts to the group of tubular adenoma patients (p = 0.011). This very important finding highlights that MACC1 blood levels can be used to distinguish patients with neoplastic transformation before reaching the carcinoma state. The difference between normals and hyperplastic patients’ plasma MACC1 transcripts was not statistically significant in our study (Fig. 3). This finding might be due to a lack of expression of MACC1 at the hyperplastic level or to the small number of such cases (n = 15) included in this comparison. Thus, validation of our findings in independent patient cohorts, ideally arising from African American as well as other e.g. Caucasian populations, is desired. It is worth noting however the ROC curve for MACC1 transcripts for HPP vs. Normal was fair (AUC = 0.60 with a sensitivity of 60 % and a specificity of 71 %). These values were much more significant in the case of adenoma vs. normal comparison (AUC = 0.67 with a sensitivity of 67 % and a specificity of 71 %). These findings highlight the potential use of MACC1 plasma transcripts as a diagnostic tool to stratify patients with potential metastatic colonic lesions. This non-invasive quantitative detection overcomes the limitation of snapshot analyses in tissues. We found higher levels of circulating MACC1 transcripts in CRC patients including those of early stages linked to shorter survival [28]. Circulating MACC1 transcripts have also been used as biomarker in the context of non-small cell lung cancer and gastric cancer [29, 30]. However, its use at precancerous stages has not been reported before our study.

In the future, the multimarker combinations of circulating MACC1 transcripts with further blood-based mRNA markers [43, 44] as well as with blood-based miRNA markers [45] might be beneficial for the improvement of diagnosis and prognosis of colon adenoma patients.

## Conclusion

We report a significant MACC1 protein expression induction in colon adenoma patients’ tissue and a significant induction of circulating MACC1 transcripts in colon adenoma patients’ blood, compared to lesion-free volunteers. Our findings might be very relevant for the use of MACC1 for prognostication of unfavorable tumor progression and metastatic potential of colorectal lesions.

## Abbreviations

C %: cytoplasmic percentage
CI: confidence interval
CRC: colorectal cancer
HPP: hyperplastic polyp
ICC: intraclass correlation coefficient
IHC: immunohistochemistry
MACC1: metastasis-associated in colon cancer 1
ROC: receiver operating characteristics curve
qRT-PCR: quantitative real time reverse transcriptase-polymerase chain reaction
STR: short tandem repeat
TA: tubular adenoma
TMA: tissue microarray
TV: tubulovillous adenoma
